# The Effect of Green Tea Beverage on Blood Cardiometabolic Risk Biomarkers in Dyslipidemia Subjects

**DOI:** 10.1002/fsn3.70415

**Published:** 2025-06-17

**Authors:** Tikhamporn Pormlikul, Nattira On‐Nom, Uthaiwan Suttisansanee, Piya Temviriyanukul, Dunyaporn Trachootham, Chanakan Khemthong, Niramol Muangpracha, Sirinapa Thangsiri, Khemika Praengam, Chaowanee Chupeerach

**Affiliations:** ^1^ Master of Science Program in Toxicology and Nutrition for Food Safety, Institute of Nutrition Mahidol University Salaya Phutthamonthon, Nakhon Pathom Thailand; ^2^ Institute of Nutrition Mahidol University Salaya Phutthamonthon, Nakhon Pathom Thailand

**Keywords:** bottled tea drink, catechins, gene expression, oxidative stress, randomized double‐blind placebo‐controlled trial

## Abstract

Green tea bioactive compounds show promise as a potential functional food to improve blood lipid profiles and ameliorate cardiovascular diseases. To determine the effect of 6 weeks of green tea compared to the placebo beverage consumption on blood cardiometabolic biomarkers. A randomized double‐blinded placebo‐controlled trial was conducted on dyslipidemia participants and randomly allocated in green tea beverage (*n* = 30) and placebo beverage (*n* = 30) groups. Sixty dyslipidemia adults with age 30–60 years old participated. Change in blood parameters, including lipid profiles, liver and kidney functions, oxidative stress and antioxidant markers, gene expressions, and anthropometric parameters, was measured. After 6 weeks of green tea intervention, blood total cholesterol and low‐density lipoprotein cholesterol (LDL) decreased in the green tea group by 4.96% and 7.98%, respectively. Plasma malondialdehyde (MDA), a lipid peroxidation marker, decreased, whereas antioxidant capacities measured by the ferric ion reducing antioxidant power (FRAP) and oxygen radical absorbance capacity (ORAC) assays were maintained in the green tea beverage group. The lipid‐related gene expression of peripheral blood mononuclear cells (PBMC) was observed by real‐time polymerase chain reaction. The LDL receptor gene was upregulated in the green tea beverage group. The liver and kidney function tests showed no alterations among the subjects in the green tea beverage and placebo groups. Six weeks of green tea beverage intervention showed potential as a treatment to reduce cardiometabolic risk in dyslipidemia subjects.

**Trial Registration:** Thai Clinical Trial Registry: TCTR20250104003

## Graphical abstract

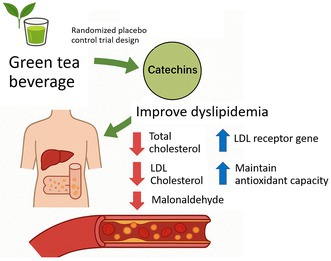

**Abbreviations**
ABCA‐1: ATP‐binding cassette transporter A1
ALP: alkaline phosphatase
ALT: alanine transaminase
AST: aspartate aminotransferase
BUN: blood urea nitrogen
FRAP: ferric ion reducing antioxidant power
HDL: high‐density lipoprotein cholesterol
HMGCR: 3‐hydroxy‐3‐methyl‐glutaryl‐coenzyme A reductase
hs‐CRP: high‐sensitivity C‐reactive protein
LDL: low‐density lipoprotein cholesterol
LDL‐R: low‐density lipoprotein receptor
MDA: malondialdehyde
ORAC: oxygen radical absorbance capacity
PBMC: peripheral blood mononuclear cell

## 1 Introduction

Cardiovascular disease (CVD) is a significant global health concern, encompassing various conditions affecting the heart and blood vessels such as coronary artery disease, stroke, hypertension, and peripheral artery disease. CVD is the leading cause of mortality worldwide, responsible for millions of deaths annually (Lindstrom et al. 2022). A major risk of CVD is dyslipidemia, which is categorized by abnormal blood lipid (fat) levels including high levels of triglycerides and LDL and low levels of HDL. These dyslipidemic conditions encourage the development of atherosclerotic plaques in the arteries, resulting in an increasing risk of CVDs (Wazir et al. 2023). One strategy for ameliorating the onset of dyslipidemia is the consumption of food with bioactive ingredients that have hypolipidemic properties.

Green tea is derived from the leaves of *Camellia sinensis* and has been consumed for centuries in various cultures, including China, Japan, and European countries. Green tea is renowned for its rich antioxidant content from natural compounds, particularly (Hinojosa‐Nogueira et al. 2021; Zhao et al. 2022). Many types of catechins are contained in green tea, such as epigallocatechin gallate (EGCG), gallocatechin gallate (GCG), epigallocatechin (EGC), epicatechin gallate (ECG), epicatechin (EC), and catechin gallate (C). Green tea also contains caffeine and other natural compounds.

Epidemiological studies have suggested an inverse association between green tea consumption and the risk of noncommunicable diseases, including CVDs, obesity, type 2 diabetes, hypertension, and cancer (Liu et al. 2024; Xu et al. 2020). Green tea combats CVDs by hypocholesteremic activity via molecular mechanisms, including reduced LDL cholesterol synthesis, cholesterol absorption from the intestine, antioxidant activity, and anti‐inflammatory activity (Bursill et al. 2001, 2007; Suzuki‐Sugihara et al. 2016).

In a clinical study, 10 cups/day of brewed green tea consumption reduced total cholesterol and LDL levels in healthy subjects after 3 weeks (Tokunaga et al. 2002). With 583 mg of total catechins in green tea beverages, obese subjects showed reduced total cholesterol and LDL but not HDL after 12 weeks (Nagao et al. 2007). A 12‐week consumption of green tea beverage (780.6 mg of total catechins) improved blood lipid profiles, oxidative stress markers, and hepatoprotective activity in mild dyslipidemia subjects (Venkatakrishnan et al. 2018). A green tea extract supplement of 600 mg of total catechins reduced LDL but not HDL levels after 8 weeks (Basu et al. 2010) with similar results recorded by 625 and 800 mg of EGCG after 12 weeks and 6 months, respectively (Asbaghi et al. 2020; Maki et al. 2009).

Green tea has been associated with lipid‐lowering effects, but the optimal dosage and duration of consumption for dyslipidemic subjects are not well defined. Existing studies had varied protocols, and the amount of green tea that gave the most benefit was unclear. There is a lack of research on personalized responses to green tea consumption in dyslipidemic populations, considering oxidative stress markers, genes, and lifestyle variability.

Therefore, this study investigated the effect of 6 weeks of green tea beverage consumption on blood lipids, oxidative stress and antioxidant capacities, inflammation markers, and lipid metabolism gene expressions in Thai dyslipidemic subjects. Our data could be used as scientific evidence for proposing green tea beverages as a functional food for dyslipidemia patients.

## 2 Materials and Methods

### 2.1 Materials

The green tea beverage and placebo were developed and supplied by Oishi Group Public Company Limited. The green tea contained tea leaves and sucralose, whereas the placebo contained water. The tea flavor was adjusted to blind the subjects. Each sample was provided in a 500‐mL airtight sealed plastic bottle with cold aseptic filling. The catechins and caffeine content were measured by the HPLC method at the Department of Food Engineering, King Mongkut's University of Technology Thonburi, Thailand. The contents in the beverage were 898.1 mg of total catechin, 149 mg of EGCG, 55.9 mg of EGC, 70.6 mg of ECG, 279.5 mg of GCG, 36 mg of EC, 115.3 mg of gallocatechin (GC), 167.7 mg of catechin gallate (CG), 54.1 mg of catechin (C), 178 mg of caffeine, and 1399.7 mg GAE of total polyphenol.

### 2.2 Study Design

The trial was designed as a randomized double‐blind placebo‐controlled trial. Subjects and researchers who worked in subject allocation, data collection, and statistical analysis were blinded. The subjects were randomly assigned to the green tea and placebo beverage groups according to age and gender.

### 2.3 Ethical Approval

Informed consent was obtained from all subjects before the study began, and the study was approved by the Mahidol University Central Institute Review Board, COA. MU‐CIRB 2023/029.1603. The research conformed to all the ethical requirements of the Helsinki Declaration.

### 2.4 Sample Size

The main objective of this study was to examine the decrease in blood lipid profiles after 6 weeks of green tea or placebo beverage consumption. The sample size (*n* = 60) was decided based on the smallest significant difference in blood LDL‐C variables indicated by Venkatakrishnan et al. (2018), with *α*‐value 0.05, power 90%, and an expected dropout rate of 10%.

### 2.5 Subjects

The inclusion criteria were adults aged 30–60 who had total cholesterol > 200 mg/dL and/or LDL cholesterol > 130 mg/dL, < 189 mg/dL, or triglycerides > 150 mg/dL, < 400 mg/dL, no history of diabetes, CVDs, cancer, acquired immunodeficiency syndrome, kidney or liver disease, smoking, substance abuse, alcohol consumption, had taken no lipid‐lowering supplements or drugs within 3 months, had no weight loss of more than 3 kg within the last 3 months, and had no food allergies including tea, caffeine, or sweeteners. Exclusion criteria were pregnancy and lactation in women, abnormal CBC, liver, and kidney function test results.

### 2.6 Intervention and Study Procedure

**FIGURE 1:**
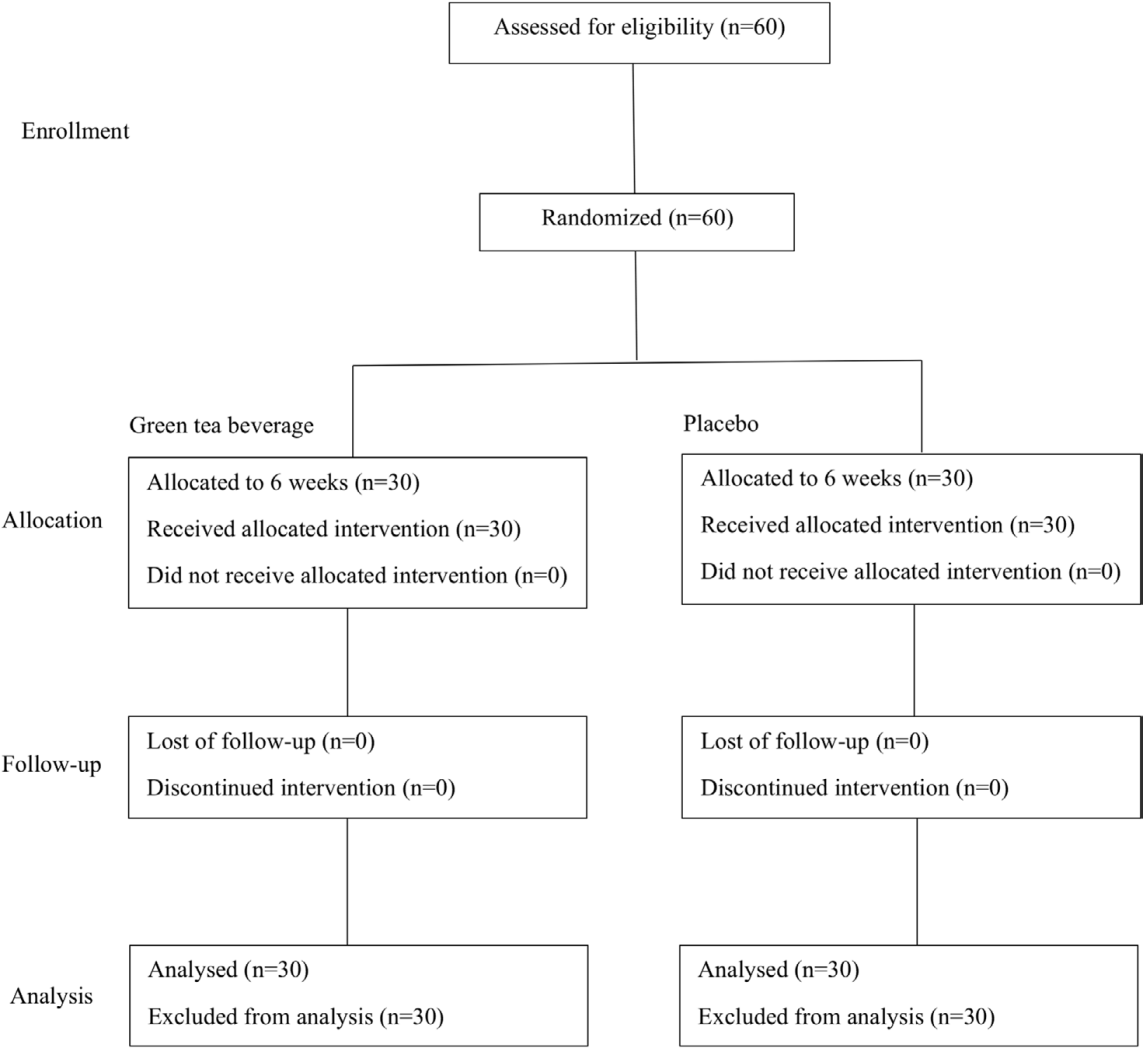
Study diagram.

The study was performed according to the Declaration of Helsinki and ICH‐GCP, as indicated in Figure 1. On the screening day, blood was drawn from the subjects for biochemical assessment to measure the anthropometric parameters and determine their medical history. The subjects were asked to maintain their lifestyles, including dietary patterns and physical activities. At the baseline experimental day, the subjects were instructed to consume two bottles containing 898.1 mg of total catechin or a placebo beverage daily for 6 weeks after lunch or dinner (Sugita et al. 2016). All the subjects were asked to follow their routine lifestyles, including dietary intake and physical activity, with no change for 6 weeks and to avoid higher dietary polyphenols. At the 3‐week follow‐up, subjects received the sample for consumption and were interviewed for any side effects. Data collected at the baseline and 6 weeks included fasting blood, blood pressure, anthropometric, and dietary assessments.

### 2.7 Outcomes

Anthropometric data including body weight, body fat percentage, and visceral fat score were measured by a Bioelectrical Impedance analyzer (BIA) (Omron Karada bodyscan, Model HBF‐375; Osaka, Japan). The body mass index (BMI) was calculated as weight (kilograms; kg) divided by height in meters squared (m^2^). For dietary intake data, 3‐day food records and 24‐h recall were applied and calculated using INMUCAL v4 software (Institute of Nutrition, Mahidol University, Thailand). For blood collection, blood samples (10 mL) were collected intravenously by a registered nurse and centrifuged at 3500 rpm (2000 *g*) for 15 min. Plasma and serum were collected in a 1.5‐mL tube and stored at −80°C until analysis.

Biochemical assessments including fasting blood glucose, insulin, and lipid profiles including total cholesterol, high‐density lipoprotein cholesterol (HDL), low‐density lipoprotein cholesterol (LDL), and triglyceride, liver function tests including aspartate aminotransferase (AST), alanine transaminase (ALT), alkaline phosphatase (ALP), and kidney function tests including blood urea nitrogen (BUN) and creatinine were analyzed at Bangkok Medical Lab Co. Ltd. (Bangkok, Thailand).

Plasma oxidative stress, antioxidant, and inflammatory markers were measured. The malondialdehyde (MDA), ferric ion reducing antioxidant power (FRAP), and oxygen radical absorbance capacity (ORAC) assays were measured by the method described by Ou et al. (2002) and On‐Nom et al. (2020), with results reported in micromolar Trolox equivalent per liter (μMTE/L). The high‐sensitivity C‐reactive protein (hs‐CRP) was analyzed at Bangkok Medical Lab Co. Ltd. (Bangkok, Thailand).

Gene expression analysis in peripheral blood mononuclear cells (PBMC) was analyzed. The PBMC from EDTA blood were isolated using a histopaque‐1077 medium (Sigma Aldrich, St. Louis, MO, USA). Total RNA was extracted using a PureLink RNA Mini Kit (Thermo Fisher Scientific, USA) according to the manufacturer's instructions. Total RNA was converted into cDNA by ReverTra Ace qPCR RT Master Mix with a gDNA remover kit (Toyobo, Osaka, Japan). The relative mRNA expression was measured by quantitative reverse transcription polymerase chain reaction (qRT‐PCR) using PowerUp SYBR Green Master Mix for qPCR (Thermo Fisher, USA) in a qPCR machine (QuantStudio 5 Real‐time PCR Systems, Thermo Fisher, USA). The relative expression was calculated using the delta delta Ct (∆∆Ct) method, and the actin gene was used as a housekeeping gene (Guevara‐Cruz et al. 2021). All the reactions were performed in duplicate. The primers are listed in Table S1.

### 2.8 Statistical Analysis

Data were presented as mean and standard deviation (SD) or standard error of the mean (SEM). The changes between the baseline and Week 6 in each subject group were analyzed using the paired *t*‐test, with mean differences between the groups tested by the Student's *t*‐test using SPSS version 19 (Chicago, USA). Statistical significance was accepted at *p* value < 0.05.

## 3 Results

### 3.1 Subjects

Sixty subjects were randomly assigned to green tea (*n* = 30) or placebo consumption (*n* = 30) and all completed the intervention. The study diagram is depicted in Figure 1. Baseline characteristics in the green tea and placebo groups are shown in Table 1. The male and female subjects were 23% and 77%, respectively. Differences in age, blood pressure, fasting blood glucose, and insulin were insignificant between the green tea and placebo groups.

**TABLE 1:** Baseline characteristics of dyslipidemia subjects.

Parameter	Placebo group (*n* = 30)	Green tea group (*n* = 30)
Male/female	7/23	7/23
Age (years)	39 ± 10	39 ± 9
Systolic blood pressure (mmHg)	122 ± 13	119 ± 15
Diastolic blood pressure (mmHg)	81 ± 9	81 ± 9
Fasting blood glucose (mg/dL)	82.30 ± 6.17	85.03 ± 7.49
Insulin (μIU/mL)	11.42 ± 9.94	10.22 ± 13.22
Total cholesterol (mg/dL)	238.03 ± 34.83	237.96 ± 29.86
Triglyceride (mg/dL)	123.73 ± 45.10	102.60 ± 39.91
HDL (mg/dL)	64.60 ± 19.89	67.00 ± 15.37
LDL (mg/dL)	148.20 ± 36.47	149.96 ± 28.39

*Note:* The data are shown as mean ± SD. No statistical differences between the green tea and placebo groups were observed at *p* value < 0.05.

Abbreviations: HDL, high‐density lipoprotein cholesterol; LDL, low‐density lipoprotein cholesterol.

### 3.2 Dietary Assessments

The dietary intake per day was observed by the 3‐day food records. Between Week 0 and Week 6, subjects in the green tea beverage group had a higher energy intake than the baseline time point, which had higher carbohydrate contents (162 ± 63 kcal), whereas no difference in energy intake was recorded in the placebo group (7 ± 63 kcal). Other macronutrients, including fat and protein intake, were not changed between the subject groups (Table S2).

### 3.3 Effect of Green Tea Beverage on Anthropometric Assessments

Anthropometric data on green tea consumption including weight, BMI, waist circumference, body fat, and visceral fat were collected and expressed in Table 2. There was no significant difference in anthropometric parameters in the placebo and green tea groups at the baseline. After 6 weeks, the subjects in the green tea group showed decreased waist circumference without significance. Male subjects showed a significant decrease in waist circumference (data not shown) but the sample was small.

**TABLE 2:** Anthropometric data between the green tea and placebo groups at Week 0 and Week 6.

Parameter	Placebo			Green tea		
	Week 0	Week 6	*p*	Week 0	Week 6	*p*
Weight (kg)	63.1 ± 11.3	63.6 ± 11.5	0.098	63.4 ± 15.0	63.2 ± 14.7	0.415
BMI (kg/m^2^)	24.5 ± 3.9	24.6 ± 3.9	0.106	24.5 ± 4.9	24.5 ± 4.8	0.970
Waist circumference (cm)	82.4 ± 11.9	82.1 ± 11.4	0.683	83.3 ± 11.6	82.1 ± 11.2	0.052
Body fat %	30.0 ± 6.5	30.2 ± 6.2	0.291	30.2 ± 7.4	30.4 ± 7.4	0.217
Visceral fat	7.2 ± 3.9	7.3 ± 4.0	0.050	7.2 ± 4.7	7.2 ± 4.6	0.758

*Note:* The data, shown as mean ± SD, express the decreased intake after 6 weeks. Paired *t*‐test before and after 6 weeks, significant with *p* value < 0.05.

Abbreviation: BMI, body mass index.

### 3.4 Effect of Green Tea Beverage on Blood Lipid Profiles

The effect of green tea consumption on lipid profiles was further investigated (Table 3). After 6 weeks, the total cholesterol and LDL‐C levels in subjects who consumed the green tea beverage decreased by 11.90 mg/dL (4.96%) and 12.30 mg/dL (7.98%), respectively. However, the consumption of green tea did not have a significant impact on triglyceride and HDL‐C levels. No changes were recorded in lipid profiles in the placebo group after the 6‐week trial.

**TABLE 3:** Blood lipid profiles between the green tea and placebo groups after 6 weeks of intervention.

Blood lipids (mg/dL)	Placebo			Green tea		
	Week 6	Week 6	Mean difference (%)	Week 0	Week 6	Mean difference (%)
Total cholesterol	238.03 ± 34.83	240.43 ± 37.77	2.4 (1.41)	237.96 ± 29.86	226.06 ± 31.79^a^	−11.90 (−4.96)*
Triglyceride	123.73 ± 45.10	120.30 ± 53.09	−3.43 (−0.76)	102.60 ± 39.91	109.96 ± 48.59	7.36 (9.69)
HDL	64.60 ± 19.89	65.13 ± 19.43	0.53 (1.53)	67.00 ± 15.37	66.10 ± 16.20	−0.90 (−1.13)
LDL	148.20 ± 36.47	150.93 ± 38.35	2.73 (3.22)	149.96 ± 28.39	137.66 ± 28.88^a^	−12.30 (−7.98)*

*Note:* The data are shown as mean ± SD.

Abbreviations: HDL, high‐density lipoprotein cholesterol; LDL, low‐density lipoprotein cholesterol.

^a^ Significant difference between Week 0 and Week 6 by the paired *t*‐test.

* Significant mean difference between the groups by the Student's *t*‐test, *p* value < 0.05.

### 3.5 Effect of Green Tea Beverage on Oxidative Stress and Inflammatory Markers

**FIGURE 2:**
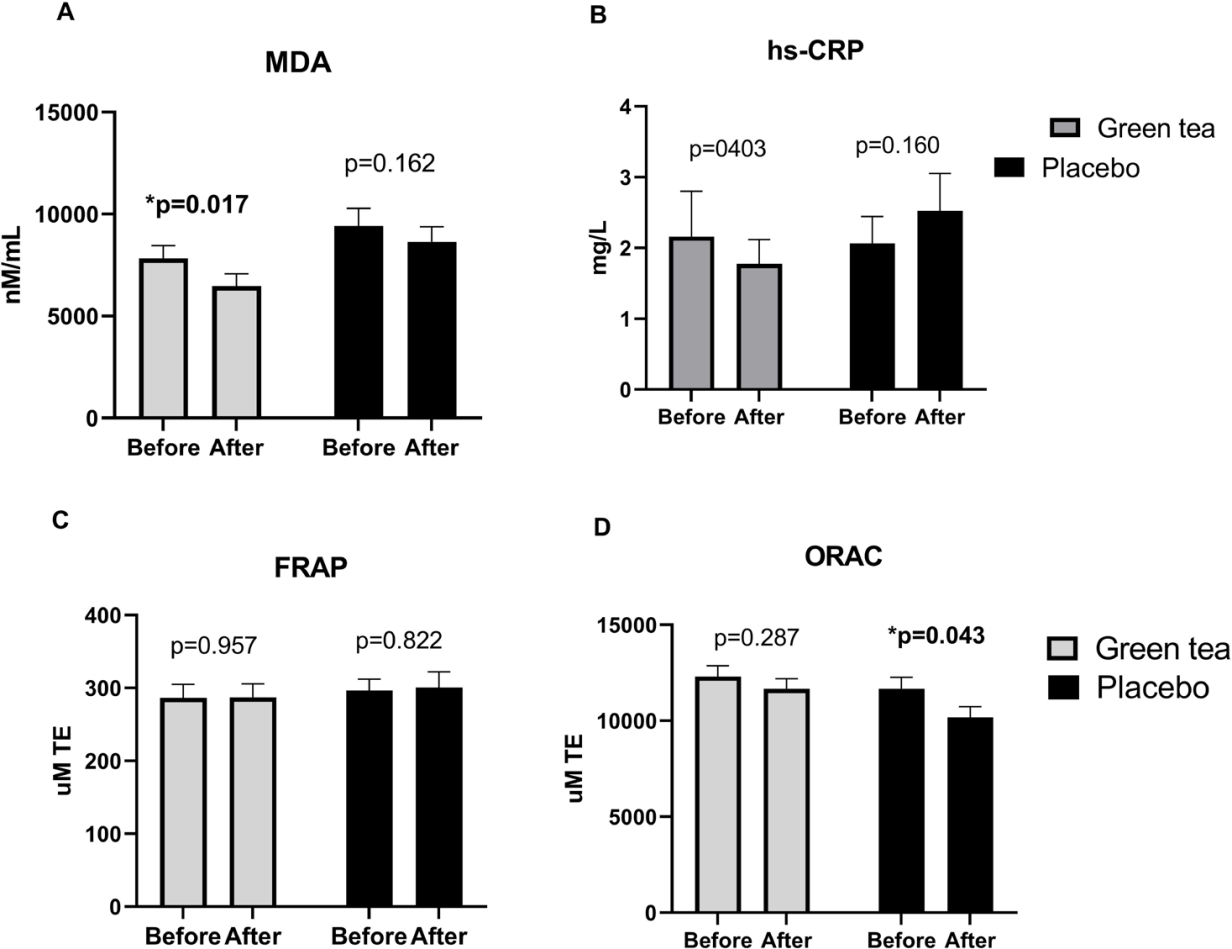
Effect of green tea beverage consumption on oxidative stress, antioxidant capacities, and inflammatory markers. Data are expressed as mean and SEM. FRAP, antioxidant capacity by the ferric ion reducing antioxidant power assay; hs‐CRP, high‐sensitivity C‐reactive protein; MDA, malonaldehyde; ORAC, antioxidant capacity by oxygen radical absorbance capacity assay. *Significant differences before and after the 6‐week trial were assessed using the paired *t*‐test, *p* value < 0.05.

Dyslipidemia has been recognized as a potential cause of oxidative stress and inflammation, often leading to cardiometabolic complications (Fukino et al. 2005). Thus, plasma cardiometabolic risk factors including oxidative stress and inflammation were measured. Malonaldehyde (MDA) was used as a marker for oxidative stress production, the FRAP and ORAC values were used as markers for antioxidant capacities, whereas hs‐CRP was employed as an indicative marker for inflammation. At the baseline, these markers were not significantly different in the placebo and green tea groups. After the 6‐week trial, MDA significantly reduced in subjects with green tea beverage consumption compared with the placebo (*p* = 0.017) (Figure 2A). The blood antioxidant capacities (FRAP and ORAC values) and inflammatory marker (hs‐CRP) showed no significant changes in the green tea subject group (Figure 2B–D), whereas a decreased blood antioxidant capacity from the ORAC assay was observed in the placebo group (*p* = 0.043).

### 3.6 Effect of Green Tea Beverage on Lipid Metabolism Gene Expression

**FIGURE 3:**
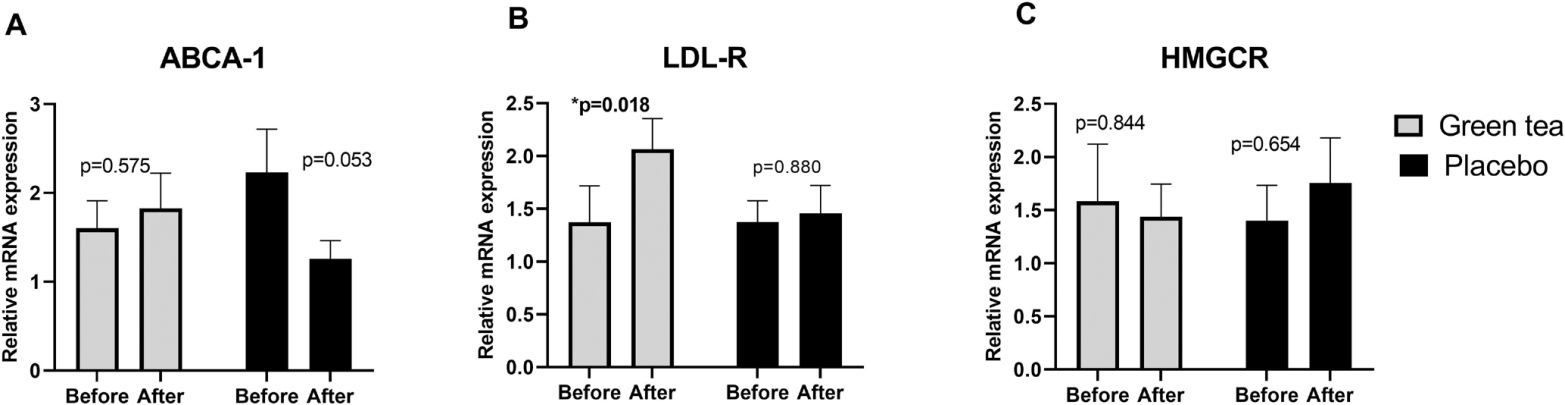
Gene expressions of the three lipid genes. Data are expressed as the mean and standard error of the mean (SEM). ABCA‐1, ATP‐binding cassette transporter A1; HMGCR, 3‐hydroxy‐3‐methyl‐glutaryl‐coenzyme A reductase; LDL‐R, low‐density lipoprotein receptor. *Significant differences before and after the 6‐week trial were assessed using the paired *t*‐test, *p* value < 0.05.

Lipid metabolism gene expression was examined to provide a molecular explanation for the hypolipidemic effect of green tea. Total RNA was isolated from the PBMC of the subjects at the baseline and after 6 weeks of the trial. Three genes were included in the analysis, including (i) ATP‐binding cassette transporter A1 (ABCA‐1), which functions to maintain cellular lipid homeostasis; (ii) low‐density lipoprotein receptor (LDL‐R), which is responsible for maintaining the plasma level of LDL; and (iii) HMG‐CoA reductase (3‐hydroxy‐3‐methyl‐glutaryl‐coenzyme A reductase [HMGCR]), which is a rate‐limiting enzyme in cholesterol (Boucher et al. 2000; Bünger et al. 2007; Guevara‐Cruz et al. 2021; Tavoosi et al. 2015). After 6 weeks of green tea consumption, the LDL‐R gene substantially increased with relative mRNA fold change: 1.3 versus 2.06 (*p* value = 0.018). Increased ABCA1 and decreased HMGR gene expressions were observed in the green tea group, but no significant differences were found. No significant changes in gene expressions were found in the placebo group (Figure 3).

### 3.7 Adverse Effects

The parameters for liver (AST, ALT, ALP, and kidney functions including BUN and creatinine) were tested to assess the adverse effects of green tea beverage consumption in the study subjects. Results showed that all the parameters were within the normal physiological ranges for all subjects at the baseline and at the end of the study (Weeks 0 and 6) (Table 4). Some minimal adverse effects were reported by two subjects as difficulty in sleeping but only for a few days during the first week of the trial (data not shown).

**TABLE 4:** Liver and kidney function test levels in the subjects after 6 weeks of the trial.

Parameter	Normal range	Placebo		Green tea	
		Male	Female	Male	Female
AST (U/L)	Male < 50, Female < 35	24.00 ± 12.6	24.54 ± 3.44	20.04 ± 2.95	20.65 ± 5.66
ALT (U/L)	Male < 50, Female < 35	28.00 ± 6.42	28.09 ± 10.15	16.5 ± 5.4	19.22 ± 9.39
ALP (U/L)	Male 40–129, Female 35–104	75.71 ± 21.66	68.00 ± 17.16	66.54 ± 16.16	70.57 ± 20.43
BUN (mg/dL)	6–20	11.6 ± 2.5	12.9 ± 2.4	11.2 ± 2.1	11.52 ± 2.7
Creatinine (mg/dL)	Male 0.67–1.17, Female 0.51–0.95	1.10 ± 0.09	1.02 ± 0.17	0.77 ± 0.09	0.71 ± 0.06

*Note:* Data are expressed as mean and SD. Paired *t*‐test, significant with *p* value < 0.05.

Abbreviations: ALP, alkaline phosphatase; ALT, alanine transaminase; AST, aspartate aminotransferase; BUN, blood urea nitrogen.

## 4 Discussion

The onset of dyslipidemia indicates a high risk for CVDs which are a leading cause of death. This study tested the lipid‐lowering effects of green tea beverage consumption to ameliorate the risk of CVDs. This was the first randomized double‐blinded placebo‐controlled trial conducted in Thailand to study how 6 weeks of green tea beverage consumption impacted dyslipidemic subjects. The consumption of 898.1 mg of total catechins in green tea beverage improved dyslipidemia blood biomarkers with decreased total cholesterol and LDL levels, improved the oxidative stress marker, MDA, maintained antioxidant capacity, and increased the LDL receptor gene expression.

Our results in dyslipidemia subjects concurred with previous clinical trials of green tea extract and beverage. In obese and overweight subjects, green tea extract (1344 mg of catechin) reduced LDL (4.8%) but not total cholesterol, triglyceride, and HDL after 6 weeks (Huang et al. 2018). Another study reported decreased serum triglyceride and free fatty acids after 12 weeks of consumption of green tea beverages (625 mg of catechin) and exercise intervention for weight loss (Maki et al. 2009). A green tea extract supplement study with 1315 mg of catechin in postmenopausal women reported reductions in total cholesterol (2.1%) and LDL (4.1%) after 12 months (Samavat et al. 2016). A lower dose of green tea drink (780.6 mg of total catechin) and longer intervention time (12 weeks) than in our study were tested in dyslipidemia subjects (Venkatakrishnan et al. 2018). After 12 weeks, blood lipid profiles including total cholesterol (6.34% reduction), LDL (4.56% reduction), triglyceride, and HDL and lipid peroxidation parameters improved in the tea‐drinking group. Results showed that the lipid profiles significantly improved at 8 weeks. Six weeks of green tea beverage consumption at 898.1 mg of catechin reduced LDL by 7.98%. These results contradicted previous studies, which found that HDL‐C levels increased or triglycerides decreased after consumption of green tea beverages or extracts. The participants in these studies were classified as overweight or obese and they had chronic complications such as hypertension or metabolic syndrome. The intervention was also extended to more than 12 weeks (Huang et al. 2018).

Green tea leaves contain a polyphenol bioactive compound that contributes to the health benefits of drinking green tea. The major polyphenols in the green tea beverage (1399.7 mg GAE) are catechins and caffeine as the bioactive compounds responsible for the synergistic effect of reducing blood lipids (Momose et al. 2016). The lipid‐lowering properties of green tea consumption include decreasing oxidative stress, cholesterol production, and fat absorption (Bursill et al. 2001; Koo and Noh 2007; Suzuki‐Sugihara et al. 2016). Drinking green tea suppresses hepatic lipase and upregulates the LDL receptor in liver cells (Samavat et al. 2016). A previous study reported that consumption of catechins; EGCG of 107–857 mg/day, reduced LDL (Zheng et al. 2011). This is comparable to drinking 2–8 cups of green tea per day, which was reported in Japan to reduce the risk of death from CVD (Saito et al. 2015). The bottled tea beverage (as in our study) contained more GCG than EGCG because of conversion during processing (Chen et al. 2001; Xu et al. 2004). GCG is an epimer of EGCG that has been assessed in a few clinical studies. Some researchers in cell culture reported that GCG showed strong antiobesity as well as EGCG (Li et al. 2017). Caffeine occurs naturally in green tea and a dose of 178.1 mg/day in beverage has been shown to impact lipid metabolism. Caffeine in green tea has also been reported to increase energy expenditure and fat oxidation in obese subjects (Hursel and Westerterp‐Plantenga 2013). Green tea containing caffeine improves anthropometric parameters including BMI and waist circumference, blood lipids (triglyceride and total cholesterol), lipid‐related genes, and protein expressions such as AMP‐activated protein kinase (AMPK), acyl‐CoA oxidase, and carnitine acyltransferase (Zhao et al. 2017). In this study, only males in the green tea group recorded a significant decrease in waist circumference. Diverse outcomes in female subjects might result from their higher carbohydrate intake. A greater number of male subjects and extension of the intervention duration clarified how drinking green tea impacted anthropometric data in a previous report (Venkatakrishnan et al. 2018).

Oxidative stress and antioxidant processes increase under dyslipidemic conditions, as contributing factors to the development of CVDs (Venkatakrishnan et al. 2018). In this study, green tea beverage reduced serum oxidative stress markers including MDA, as also reported in previous studies (Basu et al. 2013; Hirano‐Ohmori et al. 2005; Ohmori et al. 2014; Suliburska et al. 2012; Venkatakrishnan et al. 2018) (Hirano‐Ohmori et al. 2005; Basu et al. 2013; Suliburska et al. 2012; Ohmori et al. 2014; Venkatakrishnan et al. 2018). Catechins can inhibit free radical chain reactions, thereby preventing lipid peroxidation markers including MDA (Abd El‐Aziz et al. 2012). The antioxidant capacities from ORAC assays were maintained in the green tea consumption group, whereas the placebo group showed significantly lower test values. However, no change was observed in antioxidant capacity by the FRAP assay. Green tea antioxidants may not significantly contribute to the pool of reducing agents such as uric acid or vitamin C which dominate the FRAP assay. Instead, their effects may be more directly tied to scavenging specific ROS, as reflected in the ORAC assay. The lack of change in the FRAP assay, despite an increase in the ORAC assay, supports the idea that the antioxidants in green tea function in a specific and targeted manner rather than broadly increasing in all the antioxidant systems (Benzie et al. 1999; Leenen et al. 2000; Ou et al. 2002).

In this study, hs‐CRP did not change after green tea beverage consumption, possibly related to the baseline inflammatory status. The anti‐inflammatory effect of green tea may be more pronounced in individuals with elevated inflammation, such as those with metabolic syndrome or chronic inflammatory conditions (de Oliveira Assis et al. 2024). The higher carbohydrate intake in the green tea group could also dilute the change of hs‐CRP, as reported elsewhere (Giannakopoulou et al. 2024).

The gene expression in green tea drink intervention has not been studied in detail. This study investigated the effect of green tea consumption on lipid metabolism gene expressions including ATP‐binding cassette transporter A1 (ABCA‐1), 3‐hydroxy‐3‐methyl‐glutaryl‐coenzyme A reductase; HMG‐CoA reductase (HMGCR), and low‐density lipoprotein receptor (LDL‐R) using PBMC which can reflect hepatic cells for lipid metabolism (Boucher et al. 2000; Bünger et al. 2007; Guevara‐Cruz et al. 2021; Tavoosi et al. 2015). In this study, the LDL‐R gene was significantly upregulated after green tea consumption. Higher LDL‐R in the liver determines lower plasma LDL levels by uptake from the blood to the liver (Aggarwal et al. 2006). A liver cell culture study found that green tea upregulated the LDL receptor in human HepG2 cells (Bursill et al. 2001). In an animal model, green tea and polyphenol from citrus reduced lipids, especially the LDL level, by the suppression of HMGCR and an increase in the LDL‐R gene level in the liver (Bursill et al. 2007). Lower plasma lipids following the intervention, a weight reduction program, and the restriction of fat and energy intake are all related to increased expression of the LDL‐R gene in liver cells (Radler et al. 2011) and the PBMC (Mutungi et al. 2007; Øyri et al. 2019; Patalay et al. 2005). However, limited human studies have observed the LDL‐R gene expression in green tea drinks. No significant differences in the effects of green tea beverages on ABCA1 and HMGCR gene expressions were observed in this study. The expression patterns of both these genes aligned with trends reported in other dietary intervention studies (Boucher et al. 2000; Mutungi et al. 2007; Vidon et al. 2001). The ABCA1 gene plays an important role in cholesterol efflux from macrophages to HDL which may contribute to increased HDL levels (Tavoosi et al. 2015) but no changes in HDL levels were observed in this study. The HMGCR gene plays a role in cholesterol synthesis, suggesting that the lipid‐lowering effects of green tea may be mediated by enhanced LDL clearance rather than by reduced cholesterol synthesis or increased HDL levels.

The safety of green tea consumption was also studied in this investigation. Blood, liver, and kidney function parameters were within the normal range for all subjects before and after following the 6‐week intervention. The European Food Safety Authority (EFSA) reported that EGCG had the highest hepatotoxicity of catechins and was used to assess the safety of green tea catechins (EFSA 2018). Regular tea drinking, consuming not more than 5 cups of tea leaf extract per day (700 mg of EGCG), did not cause changes in liver enzymes, with no evidence of hepatotoxicity below 800 mg EGCG/day when drinking green tea extract for up to 12 months. The EFSA also reported that a daily dose of 400 mg of caffeine can be consumed without adverse effects (EFSA Panel on Dietetic Products, Nutrition, and Allergies 2015). Therefore, the doses of EGCG (149 mg) and caffeine (178.1 mg) in green tea beverages were confirmed as safe for this intervention.

This study had some limitations. The small number of male subjects should be increased, while the plasma phenolic contents, which could be helpful for lipid‐lowering mechanism explanation after green tea consumption, were not measured.

## 5 Implications for Clinical Practice

This research suggested that green tea beverage consumption with 891 mg of total catechin may offer potential benefits in reducing cardiometabolic risk markers, including its hypolipidemic effects on lipid levels, modulation of specific gene expressions, and reduction of oxidative stress after 6 weeks of intake in individuals with dyslipidemia.

## 6 Conclusions

This study is the first randomized double‐blind placebo‐controlled trial focusing on nutrigenomics to investigate the effects of 6 weeks in green tea consumption on dyslipidemia subjects. Green tea beverages containing 898.1 mg of total catechins improved lipid profiles, reduced oxidative stress parameters, and modulated lipid‐related gene expressions. Our findings highlight the potential of green tea as a functional food for reducing CVD risks, particularly in individuals with dyslipidemia.

## Author Contributions

**Tikhamporn Pormlikul:** conceptualization (supporting), data curation (supporting), formal analysis (supporting), investigation (equal), methodology (supporting), writing – original draft (equal), writing – review and editing (supporting). **Nattira On‐Nom:** conceptualization (equal), data curation (equal), formal analysis (equal), funding acquisition (supporting), investigation (equal), methodology (equal), project administration (supporting), resources (equal), software (equal), supervision (equal), validation (equal), visualization (equal), writing – original draft (equal), writing – review and editing (equal). **Uthaiwan Suttisansanee:** data curation (supporting), formal analysis (supporting), investigation (supporting), methodology (supporting), validation (supporting), writing – original draft (supporting). **Piya Temviriyanukul:** conceptualization (equal), data curation (equal), formal analysis (equal), investigation (equal), methodology (equal), resources (equal), supervision (equal), validation (equal), visualization (equal), writing – original draft (equal), writing – review and editing (equal). **Dunyaporn Trachootham:** conceptualization (supporting), methodology (supporting). **Chanakan Khemthong:** investigation (supporting), resources (supporting), software (supporting). **Niramol Muangpracha:** data curation (supporting), investigation (supporting), resources (supporting). **Sirinapa Thangsiri:** investigation (supporting), methodology (supporting). **Khemika Praengam:** investigation (supporting), methodology (supporting). **Chaowanee Chupeerach:** conceptualization (lead), data curation (lead), formal analysis (lead), funding acquisition (lead), investigation (lead), methodology (lead), project administration (lead), resources (lead), software (lead), supervision (lead), validation (lead), visualization (lead), writing – original draft (lead), writing – review and editing (lead).

## Conflicts of Interest

C.C. has received a research grant and the green tea beverage products used in this work from Oishi Group Public Company Limited, Thailand. The funders had no role in the design of the study; in the collection, analyses, or interpretation of data; in the writing of the manuscript; or in the decision to publish the results. No competing financial interests exist for the other authors. The authors have full control of all primary data.

## Supporting information


Tables S1–S2

## Data Availability

No individual data are available due to ethical restrictions.
