# Supplementary material for: The Effect of Green Tea Beverage on Blood Cardiometabolic Risk Biomarkers in Dyslipidemia Subjects
Source: Food Sci Nutr. 2025 Jun 17;13(6):e70415. doi: 10.1002/fsn3.70415 (PMC12172635; doi:10.1002/fsn3.70415)
Supplement: Supplementary file 1 — Tables S1–S2 [file FSN3-13-e70415-s001.docx]

**Supplementary materials**

**The effect of green tea beverage on dyslipidemia subjects: A randomized placebo-controlled trial**

**Table S1: Primer lists**

| **Gene** | **Sequence** | **Product size (bp)** | **Condition in qPCR** | **Primer conc in reaction**  **(nM)** | **Melting temperature** |
| --- | --- | --- | --- | --- | --- |
| *HMGCR* | Forward AGAGGCTGCAGAGCAATAGG  Reverse CATCCCATCTGCAAGGACTC | 63 | PCR profile mode consisted of hold stage 20 s at 95°C, denaturing 1 s at 95°C, annealing and extension at 20 s at 60°C 40 cycles. Run time 37 min. | 300 | 82.3 |
| *ABCA1* | Forward CACCCTTTGGCAAGTACCC  Reverse CTCAGGAGCATCATTGCTG | 86 |  | 300 | 80.8 |
| *ACTIN* | Forward AGAGCTACGAGCTGCCTGAC  Reverse CGTGGATGCCACAGGACT | 114 |  | 500 | 86.3 |
| *LDL-R* | Forward CAATGTCTCCACCAAGCTCTG  Reverse TCTGTCTCGAGGGGTAGCTG | 259 |  | 500 | 85.2 |

**Table S2:** Mean difference of dietary intake per day between before and after six weeks beverage tests in all subject groups.

| **Parameters** | **Placebo** | **Green tea** |
| --- | --- | --- |
| Energy (kcal) | 7±63 | **162±63*** |
| Carbohydrate (g) | -16.8±9 | **25±8*** |
| Sugar (g) | -6.4±5.3 | 4.9±5.7 |
| Total fat (g) | 6.0±3.6 | 4.2±4.4 |
| Saturated fat (g) | 1.71±1.2 | 1.11±1.3 |
| Protein (g) | 5.1±2.8 | 5.9±4.8 |
| Dietary fiber (g) | -1.8±0.2 | 1.4±0.8 |

Mean difference ± SEM, minus value express the decreased of intake after six weeks. *Pair T-test between before and after six weeks, *p value<0.05.
